# Screening and functional verification of potential metabolites for high fertility in sows

**DOI:** 10.3389/fnut.2026.1753880

**Published:** 2026-05-19

**Authors:** Ran Ning, Jiale Bao, Yulan Zhao, Yanlong Li, Zhekun Zhu, Shuang Cai, Xiangzhou Zeng, Xiangfang Zeng, Haiqing Sun, Fenglai Wang

**Affiliations:** 1 State Key Laboratory of Animal Nutrition and Feeding, Ministry of Agriculture Feed Industry Center, China Agricultural University, Beijing, China; 2 Beijing Key Laboratory of Biofeed Additives, China Agricultural University, Beijing, China; 3 Frontier Technology Research Institute of China Agricultural University in Shenzhen, Shenzhen, China; 4 Guangxi Yangxiang Group Company Limited, Guangxi, China

**Keywords:** amino acids, lipids, metabolites, reproductive performance, sow

## Abstract

**Background:**

Nutritional metabolism greatly influences the embryonic development and pregnancy outcomes. Exploring the metabolic differences between high and low fertility individuals plays pivotal roles in improving reproduction in mammals.

**Objective:**

The objective of this study was to explore and confirm the important metabolites influencing the reproductive potential of multiparous sows.

**Methods:**

Forty serum samples were collected from Landrace × Large White crossbred sows (3–5 parity) with high litter size (HL) (total litter size ≥ 16) and low litter size (LL) (total litter size ≤ 12) to conduct metabolomics and lipidomics, and differential analysis was applied to screen the potential important metabolite candidates. In addition, porcine trophectoderm cells (PTC) and porcine endometrial stromal cells (PEEC) were used to evaluate the effects of potential metabolite candidates on *in vitro* embryo adhesion efficiency and the expression of key genes related to trophoblast adhesion and endometrial receptivity.

**Results:**

Through integrated untargeted metabolomics and lipidomics in the serum of sows with different fertility, we identified 37 upregulated metabolites including 3-methylglutaric acid, allantoin, uridine 5’-monophosphate (UMP), asparagine, oxypurinol, oleamide, phosphatidylcholine (PC), and cholesterol ester (ChE) and 77 downregulated metabolites including cholic acid, chenodeoxycholic acid, ursodeoxycholic acid, chenodeoxycholic acid 24-glucuronide (CDCA-24G), triglyceride (TG), phosphatidylethanolamine (PE), and (O-acyl)-ω-hydroxy fatty acid (OAHFA). The altered metabolites were involved in arginine and proline metabolism, purine metabolism, sphingolipid metabolism, ether lipid metabolism, and phenylalanine, tyrosine and tryptophan biosynthesis. In addition, *in vitro* attachment models revealed that asparagine and PC (18:0_22:6) exhibited higher efficiency (*P*<0.05) in promoting embryo implantation and the underlying mechanism might involve the enhancement of cellular function related to trophoblast adhesion and endometrial receptivity.

**Conclusion:**

Our results suggest that asparagine and PC (18:0_22:6) may serve as potential key functional metabolites to enhance reproductive capacity. These findings highlight metabolic targets and potential nutritional strategies for improving fertility in mammals.

## 1 Introduction

Reproductive performance is a fundamental determinant of species survival, yet there remains considerable scope for further optimization in mammals including humans. In livestock, despite the widespread application of modern strategies such as commercial herd selection (1) and lifetime management, reproductive outcomes still fall short of theoretical potential. For example, although each sow is estimated to produce 2.5 litters per year with approximately 20 ovulations per cycle (2), the maximum number of weaned piglets per sow per year would be 52., far exceeding actual production levels. However, actual production levels fall far short of this potential (3) due to cumulative losses at multiple reproductive stages. Common limiting factors include sow mortality (typically around 10%) (4, 5), fertilization rate (85–90%) (6), farrowing rate (70–90%) (7, 8), and piglet preweaning mortality (10–15%) (9, 10). These losses not only limit livestock productivity and economic returns, but also compromise animal health and welfare, increase resource consumption, and challenge the sustainable development of animal husbandry (11–13). Similarly, the reproductive capacity of humans faces increasing challenges, including declining fertility rates (14) and an increasing prevalence of reproductive health problems (15). Taken together, these observations highlight the importance to clear biological factors governing reproductive performance and to develop effective strategies for its improvement in mammals.

Increasing evidence has demonstrated that nutrients associated with amino acid and lipid metabolism play critical roles in reproduction (16–19). In humans, metabolic disorders of these nutrients in serum or follicular fluid have been associated with conditions such as polycystic ovary syndrome and diminished ovarian reserve. Consistent with these observations, Fletcher et al. (20) revealed that glycine, carnitine, glycerophospholipids were identified as the most discriminatory features in separating high reproductive potential and infertile sows based on their serum metabolome. In a separate study, abnormal lipid metabolites including the accumulation of nervonic acid were found in sows with low embryo survival rates (21). Critically, dietary supplementation with beneficial metabolites has been shown to improve reproductive outcomes. For instance, dietary tryptophan supplementation in sows from weaning to day 28 of pregnancy increases litter weight and born alive per litter through the regulation of steroid hormones and the intestinal microbiota (22, 23), while maternal N-carbamylglutamate supplementation—arginine precursor—during early pregnancy reduced embryonic loss and enhanced live litter size (24, 25). Supplementation with short chain fatty acids (e.g., butyrate) during gestating or lactation shortens the weaning-to-estrus interval and improves subsequent farrowing rate and litter size in sows (26, 27). Based on these observations, we hypothesize that the nutritional metabolic status of sows is an essential determinant of reproductive performance, and that specific serum metabolites represent viable targets for nutritional intervention to enhance embryo implantation and fertility outcomes.

In this study, we compared metabolic differences between sows with HL and LL to identify potential metabolites for high fertility in the serum of sows via metabolomic and lipidomic analysis. In addition, we conducted *in vitro* embryo adhesion and gene expression experiments to investigate their effects on trophoblast development and endometrial receptivity. These screened important metabolites as nutritional instruction might provide further insight into promoting reproductive performance in mammals.

## 2 Materials and method

### 2.1 Animals and experimental design

Forty healthy sows (Landrace × Large White; 3–5 parity) were obtained from Ningqiang Sano Modern Animal Nutrition Co., Ltd. (Shanxi, China). They were assigned to two groups (*n* = 18 each for metabolomics; *n* = 14 each for lipidomics) based on average total litter size. HL sows had ≥ 16 total born piglets and LL sows had ≤ 12 total born across two parities. All sows received estrus synchronization and fixed-time artificial insemination to ensure consistency in follicular development, ovulation, and fertilization. The sows were transferred to farrowing house on day 108 of gestation. Each sow was housed in one individual farrowing cage (1.8 m × 2.4 m) in a temperature and humidity controlled facility under a 16-h light/8-h dark cycle. They were restrictively fed from day 0 of gestation to the day of farrowing. The chemical composition and nutrient level of gestating diet were shown in Table 1. The sows were fed a 2.50 kg/d diet from d 0 to d 90 and a 3.10 kg/d diet from d 91 to farrowing during pregnancy. Sows were fed twice daily at 7:00 a.m. and 2:00 p.m., with no leftovers ensured. All the sows had free access to water and basal diet during the whole lactating period. Water was automatically provided by nipple drinker and its quality requirements comply with Sanitary Standard for drinking water (GB5749-85). Following an 8-h fasting period immediately following farrowing, 5 mL of blood was collected from the jugular vein into sterile tubes. To minimize suffering and stress, sows were moved to sow handling equipment with exposed ears. After wiping with an alcohol swab, blood samples were collected and the wound would be pressed down using a cotton swab to ensure hemostasis. Also, we need to briefly observe whether the sows’ behavior was normal. The blood was allowed to clot at room temperature for 4 h. The serum was separated by centrifugation (3,000 × g, 10 min, room temperature) and stored at −80°C for subsequent metabolomic and lipidomic analyses. Anesthesia and euthanasia were not used in these processes. This study was conducted under the Chinese guidelines for animal welfare. All procedures were approved by the China Agricultural University Animal Care and Use Committee (Aw30705202-1-1).

**TABLE 1:** Chemical composition and nutrient level of gestating diet (%, as fed basis).

Items	Content
Corn	81.43
Soybean meal	15.62
Dicalcium phosphate	1.05
Limestone	1.00
Salt	0.40
Premix^1^	0.50
Total	100.00
Nutrient level	
Dry matter	89.08
Crude protein	14.86
Ether extract	2.26
Crude fiber	3.46
Neutral detergent fiber	9.52
Acid detergent fiber	3.08
Crude ash	3.45
Gross energy, MJ/kg	16.10

^1^Premix provided per kilogram of complete feed: 12,000 IU of vitamin A, 3,000 IU of vitamin D_3_, 20 IU of vitamin E, 1.8 mg of vitamin K_3_, 2.0 mg of vitamin B_1_, 6.0 mg of vitamin B_2_, 4.0 mg of vitamin B_6_, 3,000 mg of choline, 0.02 mg of vitamin B_12_, 26.0 mg of niacin, 18.0 mg of pantothenic acid, 3.2 mg of folic acid, 0.4 mg of biotin, 400 mg of Fe, 20 mg of Cu, 100 mg of Zn, 50 mg of Mn, 1.2 mg of I, 0.30 mg of Se, 8.0 g of Ca, 0.8 g of P, 5.6 g of sodium chloride, and 0.05% of lysine.

### 2.2 Cell culture

The PTC (Catalogue number: BFN60808646) and PEEC (Catalogue number: BFN60808569) were purchased from Qingqi Biotechnology Development Co., Ltd. (Shanghai, China) and cultured as described in previous studies (28, 29). The thawed PEEC and PTC cells were cultured in Dulbecco’s modified Eagle’s medium/high glucose medium supplemented with 10% fetal bovine serum, and 1% gentamicin-penicillin-streptomycin at 37 °C and 5% CO_2_ in a humidified atmosphere. Cells at passages 3–5 were used for all experiments to maintain cell characteristics and viability.

### 2.3 Cell vitality analysis

The optimal concentration of different metabolites for treating PTC and PEEC were screened via a Cell Counting Kit-8 (CCK-8) (Dojindo, Japan) according to the manufacturer’s protocols.

### 2.4 Cell adhesion assay

Embryo adhesion requires the coordinated action of PTC and PEEC. We first treated PTC with screened metabolites to adhere PEEC and then treated PEEC with these metabolites to adhere PTC to explore their effects on target cells. After starvation, cells were treated with the screened metabolites at the optimal concentrations for 24 h. Then, the cells were stained with carboxyfluorescein diacetate, succinimidyl ester (CFDA-SE, Beyotime, Shanghai, China) for 1 h and plated into other cell monolayers in 6-well cell culture plates for another hour. After removing the cultured medium, each well was captured via a fluorescence microscope to obtain a green fluorescence image. The integrated fluorescent density of each image was calculated as grayscale value using ImageJ software to determine the relative cell adhesion ratio.

### 2.5 Metabolite extraction and LC-MS/MS method for metabolomic analysis

Serum (100μL) was mixed with 400μL pre-chilled methanol (−80°C) to precipitate proteins. After incubation at −80°C for 1−2 h, the samples were centrifuged (14,000 × g, 10 min, 4−8°C), and the supernatants were collected, dried under N^2^, and stored at −80°C. The dried samples (HL, *n* = 18; LL, *n* = 18) were analyzed using a Q Exactive HF Orbitrap mass spectrometer with Vanquish UPLC (Thermo Fisher Scientific, United States). Metabolites were identified via TraceFinder 3.2 using accurate mass and MS/MS spectral matching (10 ppm precursor, 15 ppm fragment). A total of 1,836 metabolites were detected, including nitrogenous compounds in positive mode and carboxylate-rich compounds in negative mode. Relative quantification was based on chromatographic peak areas with a 0.25 min retention time tolerance.

### 2.6 Lipid extraction and LC-MS/MS method for lipidomic analysis

Serum (100μL) was mixed with 300μL methanol, 1 mL MTBE, and 250 μL ddH_2_O. The mixture underwent three cycles of incubation and vortexing, followed by centrifugation (8,000 rpm, 15 min, room temperature). The upper organic phase was collected, dried under N2, and stored at −80 °C. Dried samples (HL, *n* = 14; LL, *n* = 14) were analyzed using a QE Plus Orbitrap mass spectrometer coupled to a Vanquish UHPLC system (Thermo Fisher Scientific, United States). Lipids were identified and quantified with LipidSearch 4.2 using MS/MS matching. The mass tolerance was set at 8 ppm for the precursors and 10 ppm for the fragments. Lipids with chromatographic peak areas > 5E6 were considered confidently identified. A retention time shift of 0.25 min was allowed for relative quantification.

### 2.7 RNA extraction and real-time PCR analysis

RNA was extracted from PTC and PEEC treated with the screened metabolites for 24 h using TRIzol RNA Isolation Reagents (TaKaRa, Japan), chloroform, isopropanol, and ethanol according to the manufacturer’s protocols. The total RNA quality and quantity were determined by a Nanodrop system (Thermo Scientific, United States). Reverse transcription was conducted by using the PrimerScript RT Reagent Kit (TaKaRa, Japan). The specific primers employed for real-time PCR were designed using Primer Premier 6.0 software, and the sequences of these primers were shown in Table 2. cDNA was amplified to quantify gene expression via real-time PCR using gene-specific primers and SYBR Green (TaKaRa, Japan). Gene expression changes were analyzed using the 2^–ΔΔ^*^Ct^* method, and the expression levels were normalized to those of β-actin.

**TABLE 2:** Primers used in this study.

Genes	Primer sequence (5′–3′)	Size (bp)
*β-actin*	F: CTGAGAGGGAAATCGTGCGT R: AGGAAGGCTGGAAAAGAGCC	186
*aV*	F: CCCGGATTCTTGTTGCCTCT R: CCGGTGAGAAGACCAGTCAC	222
*β3*	F: ATCACGGAACCGAGATGCTC R: ATGGGTCTTGGCATCAGTGG	130
*B6*	F: TGGATGACGACCTCAACACG R: CCACAAAAGAGCCAAAGCCC	102
*CDX2*	F: TCACCATTCGGAGAAAGGCC R: GACACTTCTCAGAGGGCCTG	197
*TEAD4*	F: AAAAGCCCGTGAGATCCAGG R: CGGCTGGACAGTGTAGGTTT	244
*OCT4*	F: CGAGGAGTCCCAGGACATCAA R: TCAAAACGGCAGATGGTCGT	165
*EOMES*	F: AACACCTTACCTCAAGCCCG R: TTTGTTGGTCCCAGGTTGCT	147
*KAT8*	F: AGACAGTGAAGGATGCCGTG R: CTTCACCTTGGTGATCGCCT	194

### 2.8 Statistical analysis

Omics data were normalized prior to analysis to approximate a normal distribution. Metabolite features containing more than 50% missing values are excluded. For the remaining features, missing values are imputed with one-fifth of the minimum detected value for that feature. Metabolomic data were processed using MetaboAnalyst 6.0^1^ and an online bioinformatics platform^2^ for Kyoto Encyclopedia of Genes and Genomes (KEGG) pathway enrichment visualization. Orthogonal partial least squares discriminant analysis (OPLS-DA), cross-validation, heatmaps, and volcano plots were generated in R (v4.3.1) using the ggplot2, ropls, tidyverse, rstatix, corrplot, heatmaps, and ggrepel packages. For heatmap generation, hierarchical clustering was applied to columns (samples) using correlation distance and the average linkage method; rows (metabolites) were not clustered to preserve the original feature order. The data matrix was scaled by column prior to clustering. Additional figures were prepared with GraphPad Prism (v9.3.1, GraphPad Software, La Jolla, CA, United States). Significant differences in metabolites were defined by fold change > 1.2 or < 0.77 and *P* < 0.05. Cell experiments were performed in at least six independent replicates, with the data presented as the means ± standard error of the mean (SEM) unless otherwise stated. Other data were analyzed using *t*-tests by Statistical Product and Service Solutions (SPSS) software, with *P* < 0.05 considered statistically significant.

## 3 Results

### 3.1 General reproductive parameters of HL and LL sows

The backfat thickness and reproductive performance in HL and LL groups were presented in Table 3. Apparently, litter size and weight of present (16.94 ± 0.81 vs. 11.39 ± 0.66 kg, 19.74 ± 0.91 kg vs. 16.69 ± 1.09 kg) and previous (18.83 ± 0.53 vs. 10.06 ± 0.60, 23.59 ± 0.50 kg vs. 13.51 ± 0.73 kg) HL sows (*n* = 20) were both significantly higher than LL sows (*n* = 20) though they have similar parity (*P* < 0.01). The comparison of individual birth weight of piglets was opposite in HL (1.25 ± 0.03 kg, 1.28 ± 0.03 kg) and LL (1.49 ± 0.05 kg, 1.37 ± 0.08 kg) groups of previous parity (*P =* 0.01) and present parity (*P >* 0.05) which is well consistent with physiology.

**TABLE 3:** Backfat thickness, litter size and piglet weight of HL and LL sows (*n* = 20).

Item	LL	HL	SEM	*P-*value
Parity	3.28	3.11	0.10	0.39
Previous total litter size	11.39^a^	16.94^b^	0.70	<0.01
Previous live litter size	11.17^a^	15.56^b^	0.59	<0.01
Previous litter weight, kg	16.69^a^	19.74^b^	0.75	0.04
Previous piglet weight, kg	1.49^b^	1.25^a^	0.05	<0.01
Present total litter size	10.06^a^	18.83^b^	0.84	<0.01
Present live litter size	9.78^a^	17.67^b^	0.75	<0.01
Present litter weight, kg	13.51^a^	23.59^b^	0.96	<0.01
Present piglet weight, kg	1.37	1.28	0.03	0.08
Day 108 of gestating backfat thickness, mm	18.83	17.50	0.56	0.24
Weaning backfat thickness, mm	15.94	15.17	0.52	0.46
Backfat thickness loss, mm	2.89	2.33	0.26	0.30

HL, high litter size; LL, low litter size; Each group *n* = 20. Means within a row without a common lower-case letter (a,b) differ significantly (*P* < 0.05).

### 3.2 Serum differentially abundant metabolites and pathways between HL and LL sows based on the metabolomic analysis

**FIGURE 1:**
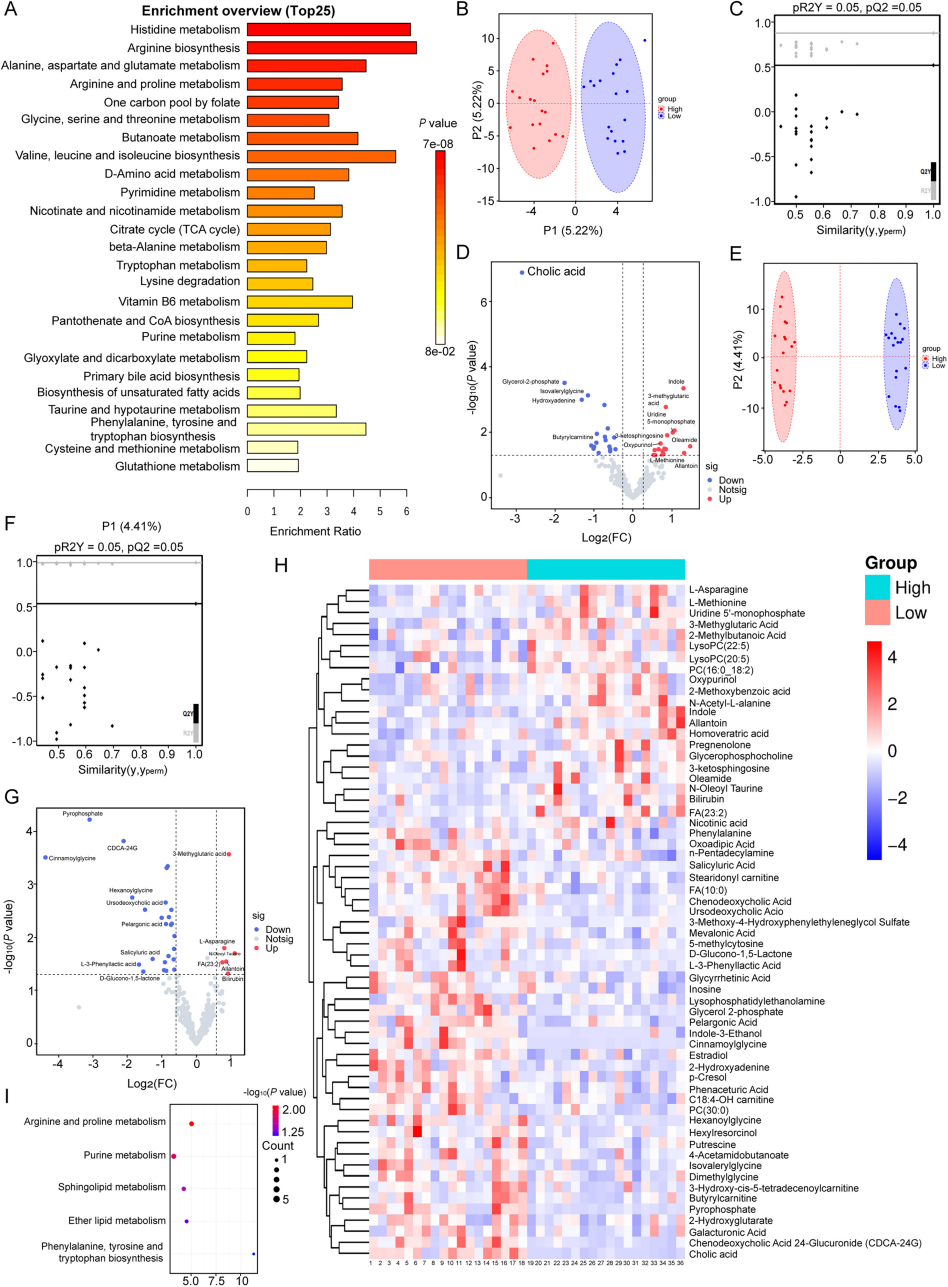
Metabolomic analysis depicting separation of serum metabolites concentrations in sows with different litter size. **(A)** Top 25 KEGG pathways enriched by all detected metabolites in sows. OPLS-DA scores plot separating sows with high and low litter size in metabolomics profiles of serum and **(B)** represents negative ions, **(E)** represents positive ions. Each group *n* = 18. **(C)** Permutation test for **(B,F)** permutation test for **(E)**. Volcano plots showing significantly different metabolites of serum in negative ion model **(D)** and positive ion model **(G)**. Fold-change threshold = 1.2, *p*-value threshold = 0.05. **(H)** Heatmap displaying metabolic profile of differentially changed metabolites. **(I)** KEGG pathway of differential metabolites identified between the two groups. **(A–G)** OPLS-DA, cross-validation, heatmaps, and volcano plots were generated in R (v4.3.1). Ellipses represent 95% confidence regions based on Hotelling’s T^2^ statistic. Significantly regulated metabolites between groups were determined by *p* < 0.05 and fold change ≥ 1.2. **(H)** KEGG pathway enrichment analysis was conducted using MetaboAnalyst 6.0 (https://www.metaboanalyst.ca) and was plotted by an online platform for visualization (https://www.bioinformatics.com.cn). HL, high live litter size (≥16); LL, low live litter size (≤11).

KEGG pathway enrichment analysis was performed on all metabolites detected by untargeted metabolomics in both HL and LL sows, and the distribution of metabolic pathways was presented in a bar chart (Figure 1A). The results showed that the identified metabolites were mainly enriched in amino acid and derivative metabolism (27.69%), lipid metabolism (18.46%), energy and carbohydrate metabolism (13.85%), vitamin, coenzyme and cofactor metabolism (10.77%), nucleotide metabolism (3.08%) and other metabolic pathways (26.15%). OPLS-DA of serum untargeted metabolomic data revealed distinct metabolic segregation between high and low litter sows in the negative ion model (Figure 1B) and positive ion model (Figure 1E). OPLS-DA analysis clearly separated the HL and LL sows, which was supported by high R^2^Y (0.877) and Q^2^Y (0.515) values in the negative ion model and high R^2^Y (0.991) and Q^2^Y (0.538) values in the positive ion model. The negative Q^2^Y intercept obtained from the permutation test in the negative ion model (Figure 1C) and positive ion model (Figure 1F) verified the reliability and robustness of the model.

Univariate analysis revealed significant metabolic differences in the serum when comparing HL and LL groups, and volcano plots were generated to show differentially abundant metabolites in the negative ion model (Figure 1D) and positive ion model (Figure 1G). A total of 31 differentially abundant metabolites were identified in the negative ion model, and 35 differentially abundant metabolites were identified in the positive ion model in the serum of HL and LL sows. Among these metabolites, the main differences included amino acids and derivatives (19), fatty acids and lipids (15), nucleotides (7), bile acids (7) and others (18). The heatmap of clustering analysis of the two groups revealed that 25 metabolites including 3-methyglutaric acid, allantoin, asparagine, oleamide, UMP, oxypurinol, indole, N-oleoyl taurine, FA (23:2), and methionine increased and that 41 metabolites including cholic acid, pyrophosphate, chenodeoxycholic acid, CDCA-24G, C18:4-OH carnitine, ursodeoxycholic acid, salicyluric acid, pelargonic acid, and Lysophosphatidylethanolamine (LysoPE) (17:0) decreased in HL serum compared with those in LL sows (Figure 1H). To comprehensively understand the potential physiological changes between HL and LL sows, we conducted KEGG pathway analysis on 66 metabolites identified in serum. As shown in Figure 1I, the enriched pathways were focused primarily on arginine and proline metabolism, purine metabolism, sphingolipid metabolism, ether lipid metabolism, and phenylalanine, tyrosine, and tryptophan biosynthesis. The original data of non-targeted metabolomics was shown in Supplementary Table 1 (the negative ion model) and Supplementary Table 2 (the positive ion model).

### 3.3 Serum differential lipid metabolites between HL and LL sows based on the lipidomic analysis

**FIGURE 2:**
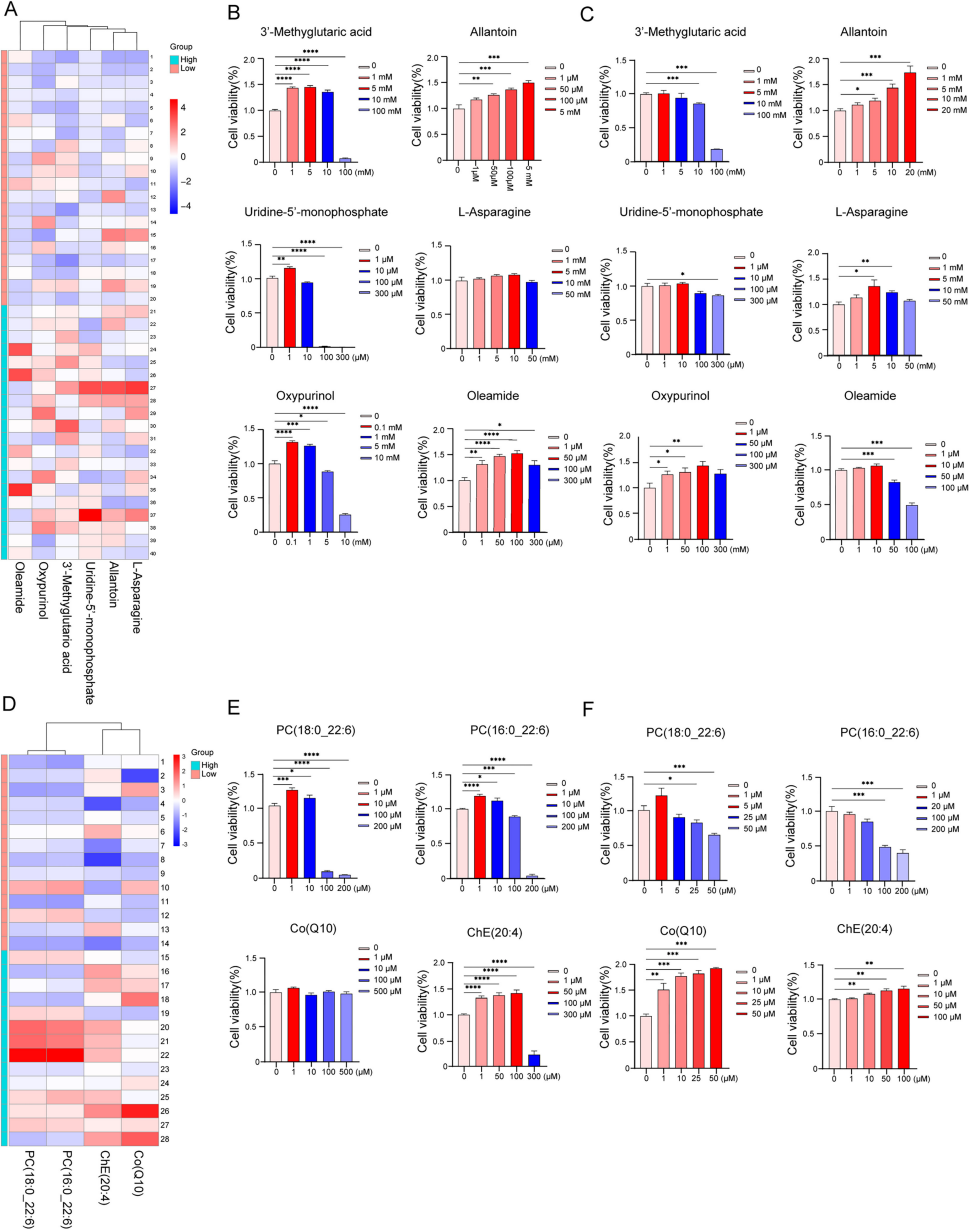
Distinct separation of lipidomics data between HL and LL groups. **(A)** Distribution of all lipid classes detected in sows. OPLS-DA scores plot separating high and low reproduction sows by lipidomic profiles measured in serum and **(B)** represents negative ions, **(E)** represents positive ions. Each group *n* = 14. **(C)** Permutation test for **(B,F)** permutation test for €. Volcano plots of lipophilic metabolites showing differential expression between HL and LL groups in negative ion model **(D)** and positive ion model **(G)**. Fold-change threshold fold change = 1.2, *p*-value threshold = 0.05. Histogram showing differentially changed lipid species. **(H)** Reds denote upregulation and **(I)** blue columns denote downregulation. **(A–F)** OPLS-DA, cross-validation, heatmaps, and volcano plots were generated in R (v4.3.1). Ellipses represent 95% confidence regions based on Hotelling’s T^2^ statistic. Significantly regulated metabolites between groups were determined by *p*-value < 0.05 and fold change ≥ 1.2. **(G,H)** Statistical significances were set by *t*-test and histograms were prepared with GraphPad Prism (v9.3.1, GraphPad Software, La Jolla, CA, United States). HL, high live litter size (≥16); LL, low live litter size (≤11).

Given the limited number of lipid metabolites identified, lipidomics was further performed. A total of 387 lipid species (28 lipid classes) were identified in the serum samples and the distribution of each class was shown in Figure 2A. Of these lipids, 26.10% were from PC, 13.7% were from sphingomyelin, 11.37% were from TG and so on. OPLS-DA of serum untargeted lipidomic data revealed distinct metabolic segregation between HL and LL sows in the negative ion model (Figure 2B) and positive ion model (Figure 2E), which was supported by high R^2^Y (0.877) and Q^2^Y (0.411) values in the negative ion model and high R^2^Y (0.990) and Q^2^Y (0.770) values in the positive ion model. The negative Q^2^Y intercept obtained from the permutation test in the negative ion model (Figure 2C) and positive ion model (Figure 2F) verified the reliability and robustness of the model.

Univariate and fold change analysis showed significant lipidic differences in serum when comparing HL and LL groups, and volcano plots were generated to show differential lipids in the negative ion model (Figure 2D) and positive ion model (Figure 2G). A total of 13 differentially abundant lipids were identified in the negative ion model and 35 differentially abundant lipids were identified in the positive ion model in serum of HL and LL sows. Among these metabolites, the main altered lipid classes included TG (26), ChE (5), PE (5), PC (4), OAHFA (3) and others (5). A heatmap was generated to visualize all differential lipids, which showed that 12 lipids including ChE (22:6), ChE (20:5), ChE (18:3), ChE (20:4), ChE (22:5), PC (16:0_22:6), PC (18:0_22:6), PC (16:0_22:5), PC (18:2_20:4) and coenzyme Q10 [Co (Q10)] increased (Figure 2H) and that 36 lipids including OAHFA (18:2_18:0), OAHFA (18:1_18:0), OAHFA (16:0_18:0), PE (18:0_22:4), PE (18:0_18:2), PE (18:0_18:1), TG (16:0_17:0_18:1), TG (17:0_18:1_18:1), TG (16:0_17:1_18:1), and TG (18:1_17:1_18:1) decreased (Figure 2I) in HL serum in comparison to LL sows. The original data of non-targeted lipidomics was shown in Supplementary Table 3 (the negative model) and Supplementary Table 4 (the positive model).

### 3.4 Effects of the upregulated metabolites in the HL sows on the *in vitro* embryo adhesion efficiency

**FIGURE 3:**
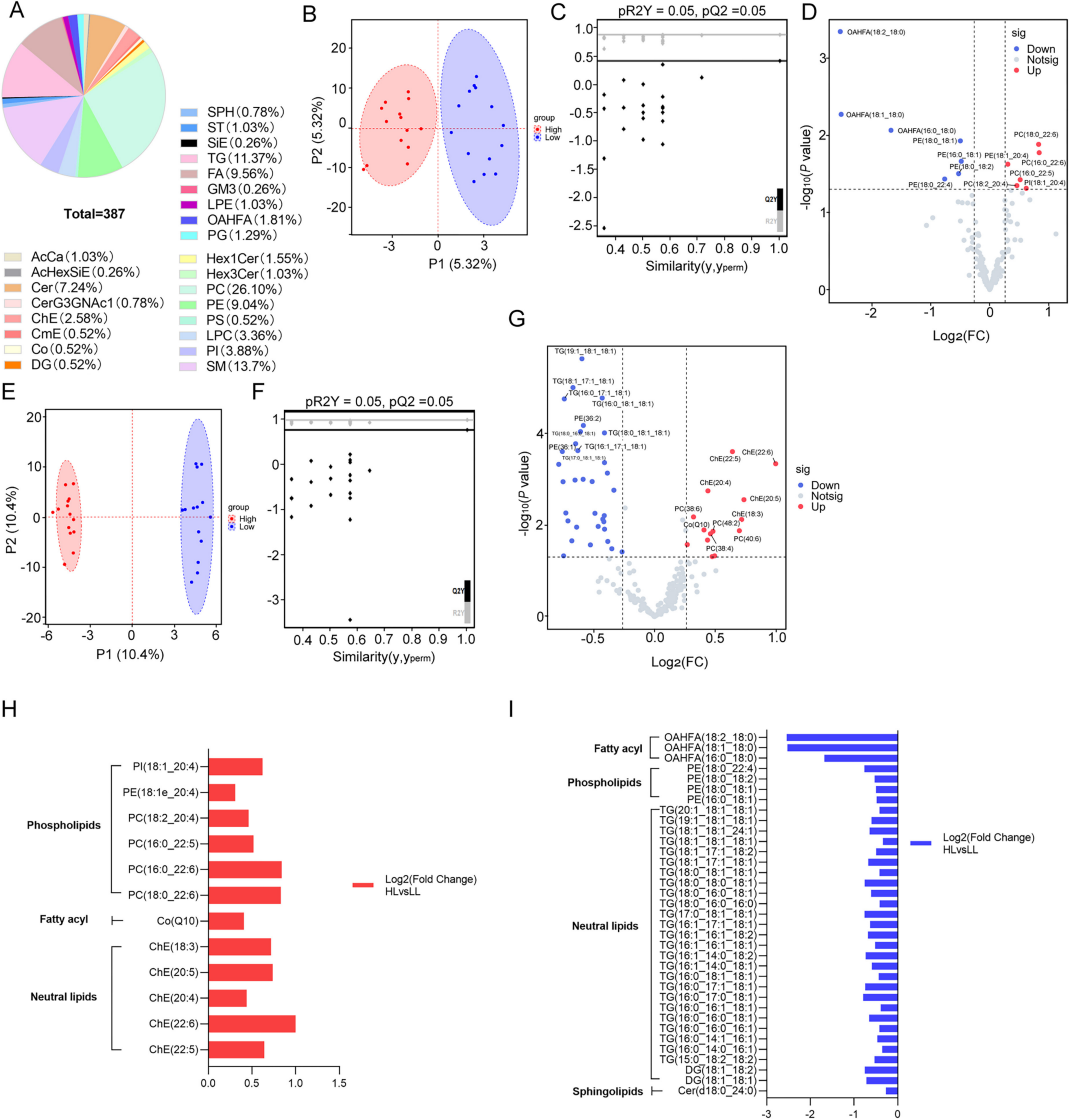
Biomarkers of metabolics predicting high litter size potential in sows. Each group *n* = 6. **(A)** Heatmap showing the expression patterns of biomarkers screened from metabolomic analysis. Cell vitality of PTC **(B)** and PEEC **(C)** treated with potential biomarkers including 3-Methylglutaric, allantoin, UMP, L-asparagine, oxypurinol and oleamide. **(D)** Heatmap showing the expression patterns of lipophilic biomarkers. Cell vitality of PTC **(E)** and PEEC **(F)** treated with potential biomarkers including PC(18:0_22:6), PC(16:0_22:6), Co(Q10) and ChE(20:4). **(A, D)** Heatmaps were generated in R (v4.3.1). **(B,C,E,F)** Histograms were prepared with GraphPad Prism (v9.3.1, GraphPad Software, La Jolla, CA, United States). Statistical significances were set by *t*-test. *P < 0.05, **P < 0.01, ***P < 0.001, ****P < 0.0001.

Heatmaps were generated to visualize ten highly expressed metabolites selected from the upregulated metabolites obtained from nontarget metabolomics (3-methylglutaric acid, allantoin, UMP, asparagine, oxypurinol, and oleamide) (Figure 3A) and non-target lipidomics [PC (18:0_22:6), PC (16:0_22:6), Co (Q10), ChE (20:4)] (Figure 3D) according to the fold change, *p*-value and availability.

The optimal concentrations of the metabolites in PTC and PEEC were exhibited in histograms. 5 mM for 3-methylglutaric acid, 5 mM for allantoin, 1 μM for UMP, 10 mM for asparagine, 100 μM for oxypurinol, 100 μM for oleamide (Figure 3B), 5 μM for PC (18:0_22:6), 1 μM for PC (16:0_22:6), 1 μM for Co (Q10) and 100 μM for ChE (20:4) (Figure 3E) were obtained according to CCK-8 proliferation assays of PTC. 1 mM for 3-methylglutaric acid, 20 mM for allantoin, 10 μM for UMP, 5 mM for L-asparagine, 100 μM for oxypurinol, 10 μM for oleamide (Figure 3C), 1 μM for PC (18:0_22:6), 1 μM for PC (16:0_22:6), 50 μM for Co (Q10) and 100 μM for ChE (20:4) (Figure 3F) were obtained according to CCK-8 proliferation assays of PEEC.

**FIGURE 4:**
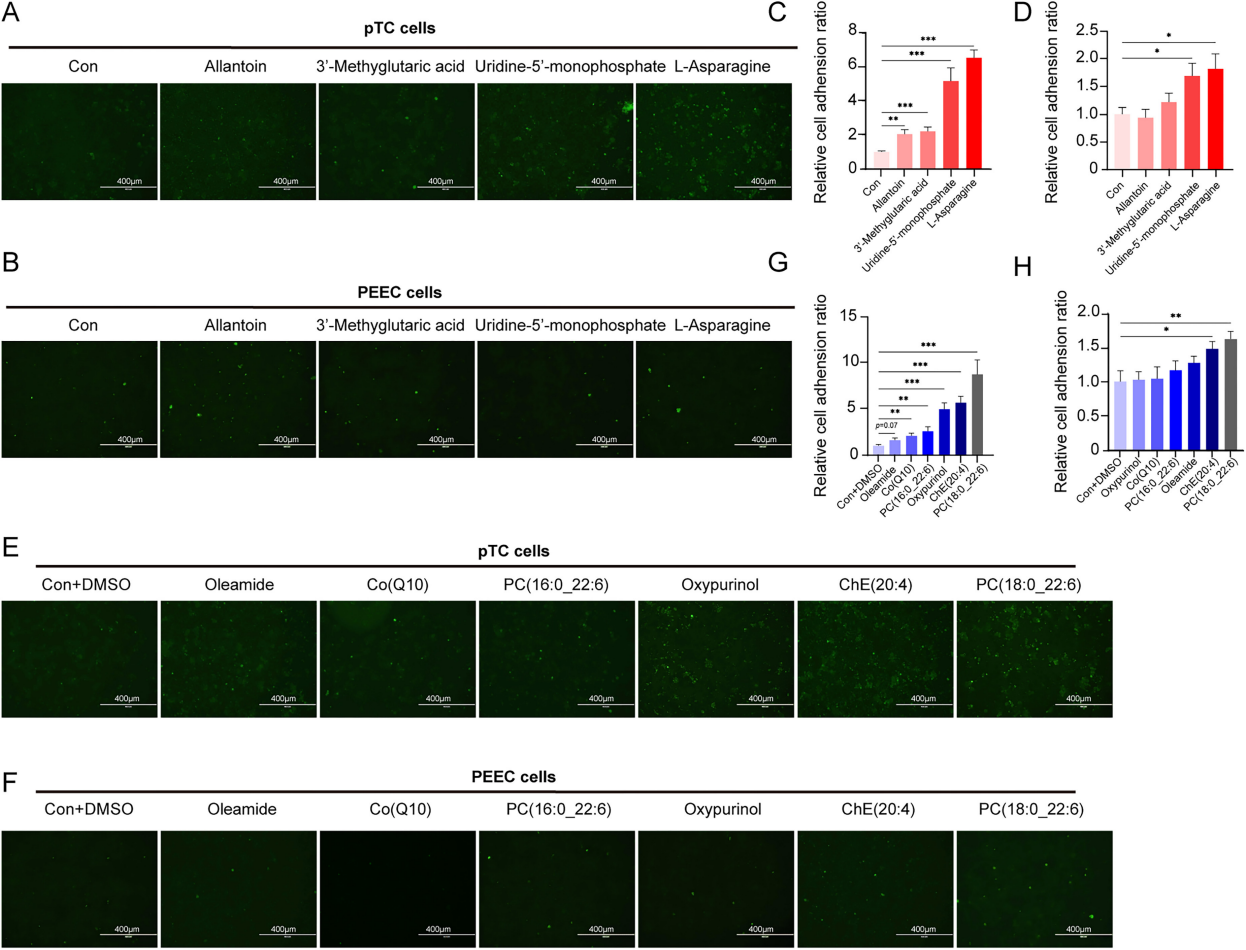
Trophoblast adhension function of biomarkers. CFDA-SE fluorescent images of PTC **(A)** and PEEC **(B)** treated with water-soluble metabolites and **(C,D)** their total grayscale value. Each group *n* = 6. CFDA-SE fluorescent images of PTC **(E)** and PEEC **(F)** treated with DMSO-soluble lipids selected from lipidomics and **(G,H)** their total grayscale value. Scale bar = 400 μm (applies to all panels). HL, high live litter size (≥16); LL, low live litter size (≤11). **(C–H)** Histograms were prepared with GraphPad Prism (v9.3.1, GraphPad Software, La Jolla, CA, United States). Statistical significances were set by *t*-test. *P < 0.05, **P < 0.01, ***P < 0.001.

The green CFDA-SE fluorescence images of PTC (Figure 4A) and PEEC (Figure 4B) treated with water-soluble metabolites and those of PTC (Figure 4C) and PEEC (Figure 4D) treated with dimethyl sulfoxide dissolved metabolites indirectly revealed the relative cell adhesion rates under different treatments. Compared with the control samples, the PTC treated with the ten screened metabolites (Figures 4C–G) and the PEEC treated with allantoin, asparagine, ChE (20:4), or PC (18:0_22:6) (Figures 4D–H) presented more intense fluorescent dots and higher grayscale values. PC (18:0_22:6) and L-asparagine each significantly enhanced a potential for embryo adhesion in both PTC and PEEC assays, with fluorescence gray values approaching the maximum among the ten screened metabolites. These two therefore warrant further investigation as potential candidates for promoting embryo adhesion.

### 3.5 Effects of the upregulated metabolites in the HL sows on the cell adhesion capability

**FIGURE 5:**
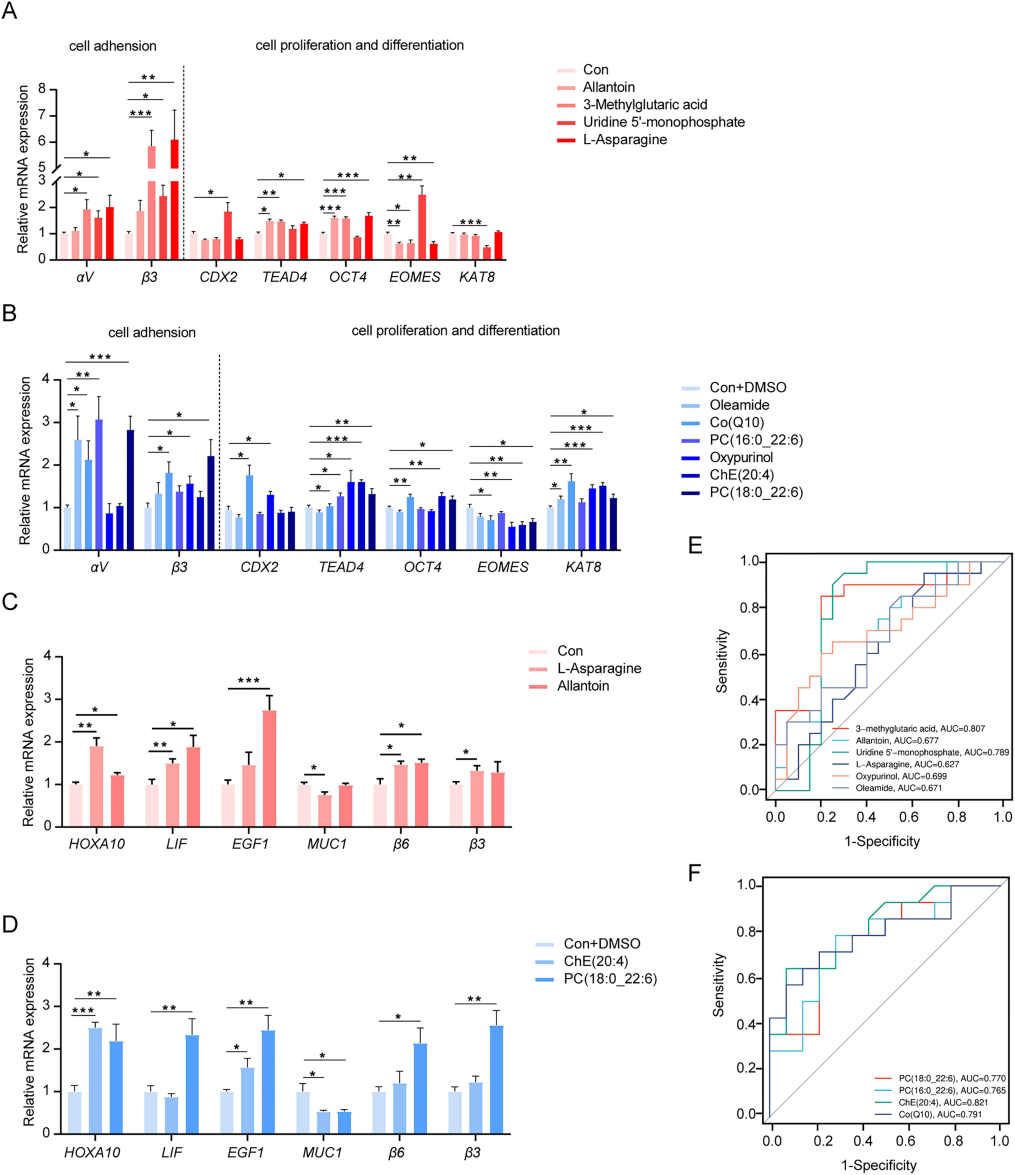
**(A,B)** Trophoblast cell adhension, proliferation and differentiation related genes expression of PTC treated with different biomarkers. **(C,D)** Endometrial receptivity related genes expression of PEEC treated with different biomarkers. The ROC curve and AUC value using screened metabolites from metabolomics **(E)** and lipidomics **(F)**. Data are presented as means ± SEM (*n* = 6 per group). **(A–D)** Histograms were prepared with GraphPad Prism (v9.3.1, GraphPad Software, La Jolla, CA, United States). Statistical significances were set by *t*-test. *P < 0.05, **P < 0.01, ***P < 0.001.

The mRNA expression of genes related to cell invasion and development in PTC (Figures 5A,B) and the mRNA expression of genes related to endometrial receptivity in PEEC (Figures 5C,D) revealed the mechanism involved in the regulation of these screened metabolites, resulting in promoting adhesion rates. Compared with those in the control group, the relative mRNA abundances of genes related to cell adhesion in PTC significantly increased (*P* < 0.05) in several treatment groups compared to the control group (Figures 5A,B). Specifically, the expression of *αV* was higher in PTC treated with 3-methylglutaric acid, UMP, asparagine, oleamide, Co (Q10), PC (16:0_22:6) and PC (18:0_22:6). Similarly, *β3* expression was elevated in the 3-methylglutaric acid, UMP, asparagine, Co (Q10), oxypurinol and PC (18:0_22:6) groups. Furthermore, we also examined the expression levels of genes related to trophoblast development. *CDX2* was significantly upregulated upon the treatment with UMP, oxypurinol, and Co (Q10) groups, whereas *Tead4* was significantly up-regulated upon the treatment with allantoin, 3-methylglutaric acid, asparagine, oxypurinol, PC (18:0_22:6), PC (16:0_22:6), Co (Q10), and ChE (20:4) groups, *OCT4* was significantly upregulated upon the treatment with allantoin, 3-methylglutaric acid, asparagine, Co (Q10), ChE (20:4), and PC (18:0_22:6) groups, *EOMES* was significantly down-regulated upon the treatment with allantoin, 3-methylglutaric acid, asparagine, Co (Q10), oxypurinol, ChE (20:4) and PC (18:0_22:6) groups and *KAT8* was significantly upregulated upon the treatment with oleamide, Co (Q10), oxypurinol, ChE (20:4) and PC (18:0_22:6) groups compared with the control group (*P* < 0.05). The mRNA expression of key genes associated with endometrial receptivity (e.g. *HOXA10, LIF* and *EGFR1*) and the expression of genes associated with cell adhesion (e.g. *β3* and *β6*) in PEEC treated with asparagine, allantoin, ChE (20:4) and PC (18:0_22:6) significantly increased (*P* < 0.05) in comparison to those of the control group (Figures 5C,D). Furthermore, treatment of PEEC with asparagine, ChE (20:4) and PC (18:0_22:6) significantly decreased the mRNA expression of *Muc1*, which was beneficial for embryo implantation (*P* < 0.05). The above results suggested that these intermediate metabolites more or less positively affected cell proliferation, development and differentiation to promote the invasion and migration ability of trophoblast and endometrial receptivity which was essential for improving embryo implantation.

### 3.6 Diagnostic performance of upregulated metabolites

A receiver operating characteristic (ROC) curve was used as a tool for evaluating the diagnostic ability of biomarkers was used to assess the sow performance of the established diagnosis model. The area under the curve (AUC), an important evaluation criterion, is positively correlated with superior diagnostic performance. The AUC values were determined to be 0.807, 0.677, 0.789, 0.627, 0.699, and 0.671 for 3-methylglutaric acid, allantoin, UMP, asparagine, oxypurinol and oleamide, respectively (Figure 5E). Similarly, the AUC values were determined to be 0.770, 0.765, 0.821, and 0.791 for PC (18:0_22:6), PC (16:0_22:6), ChE (20:4), and Co (Q10), respectively (Figure 5F). All values were higher than the AUC of 0.5 suggested that the diagnostic ability of these biomarkers was relatively accurate. Besides candidate biomarkers with high diagnostic potential including 3-methylglutaric acid, UMP and lipidomic biomarkers, it was best for others to be used in combination with other biomarkers to predict sow reproduction.

## 4 Discussion

Improving reproductive performance in mammalian animals is a necessary and eternal research, critical both to the efficiency in the livestock industry and to mammalian health. By comparing serum metabolites between HL and LL sows, we identified fertility-associated metabolic differences, including differences in arginine and proline metabolism, purine metabolism, sphingolipid metabolism, ether lipid metabolism, and aromatic amino acid biosynthesis. From these, we screened potential biomarkers such as PC (18:0_22:6) and L-asparagine, and preliminarily validated their roles in embryo adhesion and related gene expression *in vitro*. These results provide candidate indicators for sow fertility selection and suggest metabolic targets that may inform nutritional strategies and research on reproductive disorders.

In this study, relatively high levels of amino acids and their derivatives (e.g., asparagine, methionine, 3-methyglutaric acid, indole), nucleosides related metabolites (e.g., UMP, oxypurinol, allantoin), lipid metabolites including PC (≥38 carbons and ≥ 5 double bonds) and ChE (≥18 carbons and ≥ 3 double bonds) were detected in HL sows. Consistently, previous studies have reported dysregulated amino acid, nucleotide and lipid pathways in sows with impaired reproductive performance in early gestation (11, 20, 30). For the amino acids and their derivatives: Amino acids are abundant and dynamically changing in the mammalian biofluid and its turnover is known to reflects reproductive competence (31, 32). Specifically, previous studies have reported elevated methionine was associated with superior fertility (33) and favorable embryonic development (23, 34). Aspartic acid, hydrolysate of asparagine, was also shown to be lower in low-fertility sows than in high-fertility sows (30). These results are consistent with the findings in the present study. Among amino acid-related pathways, arginine and proline metabolism was the most significantly enriched. Liu et al. (35) also reported the levels of arginine, proline, and asparagine in uterine lumen fluid of gestating sows were positively correlated with advancing days of pregnancy days (from days 10 to 16), further confirming the importance of amino acids during peri-implantation period of pregnancy. For purine and pyrimidine-related metabolites: DNA and RNA are important genetic materials of life. As essential components of DNA and RNA, nucleotides also regulate genetics and reproduction. In practice, nucleotide supplementation (e.g., uridine, pyrimidine nucleosides, 5’monophosphate) can alleviate oxidative stress in sows (36), increase the viability of PTC and piglets by regulating placental nutrient transport via the mTORC1-PPAR signaling pathway (37–39). For lipids including phospholipids and cholesterol: Cholesteryl esters are an important form of cholesterol storage in cells and serve as precursors for steroid hormone synthesis (40). Li et al. found cholesterol deficiency impair embryonic development (41). Phosphatidylcholine is the most abundant phospholipid in tissues and participates in the transport and deposition of long chain polyunsaturated fatty acids. Findings from Mills et al. also indicated that gilts with higher abundance of PC (36:3) and PC (36:2) possess greater future reproductive potential (42). Therefore, the enrichment of ChE and PC in HL sows in the present study may suggest that these compounds improve reproductive performance by enhancing lipid availability to promote steroidogenesis and regulate reproductive endocrine function (43). Additionally, bile acids (e.g., cholic acid, CDCA-24G, ursodeoxycholic acid) and TG (≥51 carbons and ≥ 1 double bonds) reduced in HL sows. Li et al. found that bile acid level decreased with the progression of porcine oocyte meiosis (44), suggests that alleviating abnormally elevated circulating bile acids during gestating may improve fetal survival and piglet health (45, 46). Abnormal accumulation of triglycerides represents excessive fat deposition, which can lead to adverse pregnancy outcomes such as retarded embryonic development, impaired placental function, and compromised angiogenesis (47, 48). These findings indirectly support the results of the present study and highlight the crucial role of amino acid, nucleotide and lipid metabolic homeostasis enriched in reproduction regulation.

Among the differentially abundant metabolites identified in HL sows, we selected 10 candidates, including amino acid-related metabolites (e.g., L-asparagine, 3-methylglutaric acid, allantoin), nucleotide-related metabolites (e.g., UMP, oxypurinol, oleamide), and lipids [e.g., PC (18:0_22:6), PC (16:0_22:6), Co (Q10), and ChE (20:4)], for further functional validation using *in vitro* embryo adhesion model. Our results showed they more or less enhanced embryo adhesion potential. This finding is further supported by recent metabolomic profiling of porcine uterine luminal fluid (49). Metabolites such as asparagine and phosphorylcholine, a precursor of phosphatidylcholine increased markedly during early gestating period, supporting the hypothesis that these metabolites are not only circulating biomarkers but also active components of the uterine microenvironment that support embryo implantation. Interestingly, 3-methylglutaric acid, which had the highest AUC value, has rarely been studied associated with sow reproductive performance. 3-Methylglutaric acid is known to be involved in mitochondrial metabolism and has been implicated in reactive oxygen species generation and mitochondrial stress responses (50–52). Given that mitochondrial function is critical for oocyte quality, embryo development, and endometrial receptivity (53–55), the enrichment of 3-methylglutaric acid in HL sows may reflect enhanced mitochondrial metabolic activity rather than dysfunction that supports reproductive success. Future studies measuring mitochondrial respiration in embryo and endometrial tissues need to elucidate the specific role of this metabolite in sow fertility.

Embryonic loss is well-known to be one of the key factors limiting litter size in sows, most of the total embryonic loss occurs during the peri-implantation period (days 12–30 of gestation) (56, 57). In the commercially relevant Landrace × Large White crossbred genotype, studies have reported embryonic loss rates of approximately 30–50% during this critical window (58–61). Therefore, we hypothesized that the identified 10 metabolites may possess the potential to regulate biological processes related to embryo implantation, thereby improving pregnancy outcomes in sows. To validate this hypothesis, we evaluated the effects of these candidate metabolites on in vitro embryo adhesion using PTC and PEEC. Notably, asparagine and PC (18:0_22:6) exhibited significant effects both in PTC and PEEC, indicating that further investigation of these two metabolites is of great value. We further validated asparagine and PC (18:0_22:6) by assessing key gene expression in PTC and PEEC. Gene expression results further indicate that they are key metabolites for improving embryo adhesion and endometrial receptivity, and their underlying mechanisms warrant further investigation. For asparagine: Asparagine is transported by SLC7A5/LAT1, activating mTORC1 signaling (62, 63). Wnt/β-catenin signaling has been shown to directly regulate SLC7A5 expression during embryogenesis (64). Additionally, mTORC1 regulates the expression and membrane localization of integrins (65), while β-catenin regulates *HOXA10* and *LIF* expression in the endometrium (66). Consistent with this, our data showed that asparagine treatment increased integrin *β3*/*6*, *LIF* and *HOXA10* expression. We therefore speculate that asparagine may enhance embryo adhesion by activating mTORC1 signaling. Additionally, asparagine may influence trophoblast proliferation and lineage specification via the amino acid response (AAR) pathway (67). Studies in embryonic stem cells have demonstrated that AAR activation alters cell lineage determination (68), and asparagine may similarly affect trophoblast fate (17). Supporting this, asparagine upregulated *CDX2*, *TEAD4*, and *OCT4* while downregulating *EOMES* in PTC, suggesting a pattern indicative of maintained stemness and proliferative capacity. For PC (18:0_22:6): PC (18:0_22:6) is a phospholipid containing docosahexaenoic acid (DHA). We speculate that PC (18:0_22:6) serves as a reservoir for lysophosphatidylcholine (LPC)/lysophosphatidic acid (LPA) generation, thereby facilitating proliferation and migration of PTC (69) and trophoblast implantation (70). Additionally, DHA has been shown to activate GPR120 signaling, a pathway involved in trophoblast differentiation and function (71). GPR120 activation can enhance ERK1/2 signaling, which regulate integrin expression and endometrial receptivity (72, 73). These results were consistent with higher integrin, cell proliferation and differentiation-related expression in present study. Future studies employing pathway-specific inhibitors and siRNA-mediated knockdown approaches will be essential to validate these hypotheses and dissect the precise molecular cascades underlying effects of asparagine and PC (18:0_22:6) on function of trophoblast and endometrium. Notably, asparagine can be directly added as an additive to the diet, while PC (18:0_22:6) might can be supplied via sources such as fish oil or antarctic krill oil (74, 75). Our findings suggested that enriching sow diets with asparagine or PC (18:0_22:6) during implantation period may represent a feasible nutritional strategy to enhance litter size. Given that *in vitro* embryo adhesion assays cannot fully mimic the sophisticated microenvironment of embryo implantation *in vivo*, and differences in final litter size between HL and LL sows may arise from multiple physiological bottlenecks beyond implantation efficiency, including ovulation rate, oocyte maturation, fertilization success, and fetal resorption, further *in vivo* experiments are needed in the future to verify the predictive efficiency of the identified biomarkers, evaluate the effectiveness of these nutritional interventions, and explore more potential mechanisms underlying their beneficial effects on pregnancy outcomes.

In conclusion, we identified that asparagine and PC (18:0_22:6) were more efficient at promoting embryo implantation and the underlying mechanism might involve the enhancement of cellular functions related to trophoblast adhesion and endometrial receptivity. These findings provide critical nutritional targets for improving reproductive performance in mammals.

## Data Availability

The original contributions presented in the study are included in the article/Supplementary material, further inquiries can be directed to the corresponding author.

## Footnotes

1
https://www.metaboanalyst.ca

2
https://www.bioinformatics.com.cn
